# Supplementary material for: Causal Relationship between Aging and Anorexia Nervosa: A White-Matter-Microstructure-Mediated Mendelian Randomization Analysis
Source: Biomedicines. 2024 Aug 16;12(8):1874. doi: 10.3390/biomedicines12081874 (PMC11351342; doi:10.3390/biomedicines12081874)
Supplement: Supplementary file 1 [file biomedicines-12-01874-s001.zip › Supplementary Figure/Figure S8/Leaveoneout plot of MR analysis for TL to ubm-b-1482.pdf]

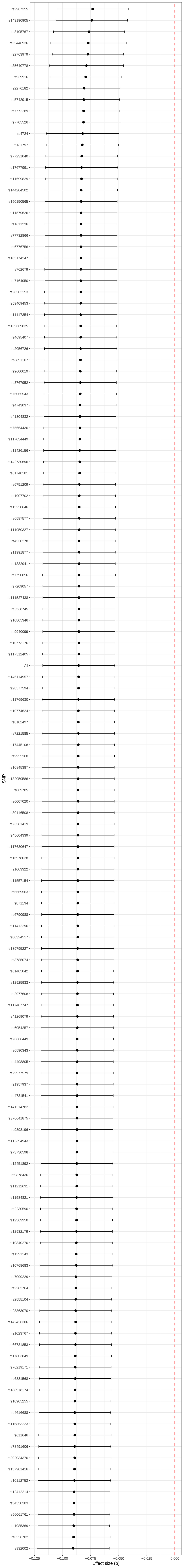
rs2967355
rs143190905
rs8105767
rs35446936
rs2763979
rs35640778
rs939916
rs2276182
rs5742915
rs7772289
rs7705526
rs4724
rs131797
rs77231040
rs17677991
rs11699829
rs144204502
rs150150565
rs11579626
rs1611236
rs77732866
rs6776756
rs185174247
rs762679
rs7164950
rs28502153
rs59409453
rs11117354
rs139669835
rs4695407
rs2056726
rs3891167
rs9600019
rs3767952
rs76065543
rs4743037
rs41304832
rs75664430
rs117034449
rs11426156
rs142730696
rs61748181
rs6751209
rs1907702
rs13230646
rs6587577
rs111950327
rs4530278
rs11991877
rs1332941
rs7790856
rs7209057
rs111527438
rs2538745
rs10805346
rs9940099
rs10773176
rs117512405
All
rs145114957
rs28577594
rs11769630
rs10774624
rs8102497
rs7221585
rs17445108
rs9955360
rs10845387
rs182059586
rs869785
rs6007020
rs80116508
rs73581419
rs45604339
rs117630647
rs16978028
rs1003322
rs11557154
rs6669563
rs871134
rs6790988
rs11412296
rs80324517
rs139795227
rs3785074
rs61405042
rs12925933
rs2977608
rs117407747
rs41269079
rs6054257
rs76666449
rs6590343
rs4498805
rs79977579
rs1957937
rs4731541
rs141214782
rs376641875
rs9398196
rs112394943
rs73730598
rs12451892
rs9878436
rs11212631
rs11584821
rs2230590
rs12369950
rs12932179
rs10840270
rs1291143
rs10768683
rs7099229
rs2282764
rs2555104
rs28363070
rs142426306
rs1023767
rs66731853
rs17803849
rs76219171
rs6881568
rs188918174
rs10905255
rs4616688
rs116863223
rs611646
rs78491606
rs202034370
rs137901416
rs10112752
rs12412214
rs34550383
rs56061761
rs1985369
rs6536702
rs932002
SNP
−0.125
−0.100
−0.075
−0.050
−0.025
0.000
Effect size (b)
